# Effect of transforming growth factor-β2 on biological regulation of multilayer primary chondrocyte culture

**DOI:** 10.1007/s10561-018-9732-z

**Published:** 2018-10-30

**Authors:** Seyed Ali Behruz Khaghani, Gunay Akbarova, Chin Fhong Soon, Gulrukh Dilbazi

**Affiliations:** 1 Faculty of Engineering and Informatics, University of Bradford, Bradford, UK; 2 Department of Genetics and Theory of Evolution, Faculty of Biology, Baku State University, Baku, Azerbaijan; 3 Biosensor and Bioengineering Laboratory, MiNT-SRC Research Center, Tun Hussein Onn University of Malaysia, Batu Pahat, Johor, Malaysia; 4 The Laboratory of Veterinary Preparations, The Veterinary Scientific-Research Institute, Baku, Azerbaijan

**Keywords:** Chondrocyte, Cytokines, TGF-β2, Gene expressions, Wound repair, Multilayer cell culture

## Abstract

Cytokines are extremely potent biomolecules that regulate cellular functions and play multiple roles in initiation and inhibition of disease. These highly specialised macromolecules are actively involved in control of cellular proliferation, apoptosis, cell migration and adhesion. This work, investigates the effect of transforming growth factor-beta2 (TGF-β2) on the biological regulation of chondrocyte and the repair of a created model wound on a multilayer culture system. Also the effect of this cytokine on cell length, proliferation, and cell adhesion has been investigated. Chondrocytes isolated from knee joint of rats and cultured at 4 layers. Each layer consisted of 2 × 10^5^ cells/ml with and without TGF-β2. The expression of mRNA and protein levels of TGF-β receptors and Smad1, 3, 4, and 7 have been analysed by RT-PCR and western blot analysis. The effect of different supplementations in chondrocyte cell proliferation, cell length, adhesion, and wound repair was statistically analysed by One-way ANOVA test. Our results showed that the TGFβ2 regulates mRNA levels of its own receptors, and of Smad3 and Smad7. Also the TGF-β2 caused an increase in chondrocyte cell length, but decreased its proliferation rate and the wound healing process. TGF-β2 also decreased cell adhesion ability to the surface of the culture flask. Since, TGF-β2 increased the cell size, but showed negative effect on cell proliferation and adhesion of CHC, the effect of manipulated TGF-β2 with other growth factors and/or proteins needs to be investigated to finalize the utilization of this growth factor and design of scaffolding in treatment of different types of arthritis.

## Introduction

Cartilage degeneration caused by injuries and congenital abnormalities is a great clinical challenge (Gillogly et al. 1998). Damage to cartilage results an incomplete wound healing and can be followed by progressive and chronic lesions (Bhosale and Richardson 2008). Cartilage can be injured by tears, general wear or injury resulting from genetic factors or overuse of tissue during sport (McIlwraith 2007).

Damage to the cartilage does initiate a reparative response (Meyer and Wiesmann 2006). However, deep cartilage defects result in the loss of non-collagenous matrix (Meyer and Wiesmann 2006) which requires complete tissue repair and in more severe cases when the lesion is in the fibrillar network and involves significant cell death the cartilage does not heal (Cubinskaya et al. 2008). In contrast if a lesion occurs in the collagenous matrix, there is a degree of regeneration. In this case treatment of cartilage by a cell therapy method and engineered tissue could enhance tissue repair (Cohen et al. 1992).

Cytokines are group of macromolecules synthesized by cells in response to immune stimuli to regulate the cell’s function (Galvani and Cowley 1992). They exert their effects on the same cells (autocrine activity) or on the neighboring cells (paracrine activity) by interacting with specific receptors (Balkwill 2000; Kermani and Pham 2001). Cytokines act as signaling proteins, and like hormones play an important role in pathophysiological and homeostatic processes such as fever, wound healing, inflammation, tissue repair and fibrosis. They are very active in regulation of cell function such as proliferation, migration, and matrix synthesis (Maitre et al. 2007). Some diseases such as degeneration of intervertebral disks could be caused by altering the expression of cytokines such as Interleukin-1 (IL-1) (Raftery and Sutherland 2002). Therefore investigation the role of cytokines in the development of diseases could be helpful in the therapy of some disorders.

Transforming growth factor-β (TGF-β) superfamily includes a large group of soluble extracellular proteins which regulate the development in both vertebrates and invertebrates (Krauss 2006; Soo et al. 2000). TGF-β family regulates the cell functions such as migration, apoptosis, proliferation and differentiation (Stoff et al. 2007; Soo et al. 2000; Spagnoli et al. 2007).

Transforming growth factor-β (TGF-β1, 2, and 3) has been implicated in the ontogenetic transition from scarless fetal repair to adult skin repair with scaring. Fibromodulin which is a member of the small leucine-rich proteoglycan family has been suggested as a biologically significant mediator of fetal scarless repair (Han et al. 2007; Cailotto et al. 2007). There is evidence that fibromodulin may be a biologically relevant modulator of TGF-β activity during scar formation (Tchetina et al. 2006). Spagnoli et al. (2007) studied the role of transforming growth factor β (TGF-β) signalling in mice lacking the TGF-β type-II receptor gene in their limbs. They found that TGF-β receptor II signalling regulates growth and differentiation factor-5 (GDF-5) and joint morphogenic gene expression (Okazaki et al. 1996). Han et al. (2007) also studied the effect of transforming growth factor-β1 on regulation of fibronectin isoform expression and splicing factor SRp40 expression during pro-chondrogenic cell line (ATDC5) chondrogenic maturation. Their report showed that the effects of TGF-β1 on fibronectin isoform splicing during chondrogenesis may be largely dependent on its effect on SRp40 isoform expression (Thorp et al. 1992).

Cailotto et al. (2007) examined the production of extracellular inorganic pyrophosphate (ePPi) in chondrocytes and the signalling pathways involved in the regulation of Ank gene expression by TGF-β. The outcome of their research shows that the TGF-β increases ePPi level, mainly by the induction of the Ank gene, which requires activation of Ca^2+^-dependent Protein Kinase C (PKC) pathways in chondrocytes (Yamashita et al. 2002). Mutations in the Ank gene were reported in autosomal dominant craniometaphyseal dysplasia and ankylosing spondylitis, supporting a key role for the Ank gene in the field of mineralizing arthropathy (Galvani and Cowley 1992; Kaiser et al. 2004).

Biological effect of TGF-β are mediated by two different serine/threonine kinase receptors, named TβRI and TβRII and important for inducing signal transduction. TβRI phosphorylates the receptor-regulated Smads (R-Smads) Smad2 and Smad3, which bind to Smad4, translocate into the nucleus and regulate gene expression in concert with other transcriptional factors (Davidson et al. 2005). Like R-Smads, the inhibitory Smad7 interacts with the activated TβRI receptor. In contrast to Smad2/3, Smad7 forms a stable association with the receptor complex and prevents receptor-mediated phosphorylation of pathway-restricted Smads, resulting in disruption of TGFβ signaling. TGF-β signalling pathway plays a critical role for maintenance of tissue homeostasis, and modification of TGF-β signaling gene expression may be a cause for articular diseases such as osteoarthritis (OA) (Oka et al. 2007). Smad3 gene mutations are associated with the pathogenesis of it (Pohlers et al. 2007). In addition, osteoarthritis is associated with modifications of TβRII and Smad7 expression (Ferguson and O’Kane 2004).

Tchetina et al. (2006) examined the effect of TGF- β2 on collagen cleavage on human osteoarthritic cartilage (Gorvy et al. 2005). Before Tchetina et al., Okazaki et al. (1996) studied the effects of transforming growth factor beta-1, beta-2 and basic fibroblast growth factor (bFGF) on articular chondrocytes obtained from immobilised rabbit knees. However, they did not use TGF-beta 3. They reported that the TGF beta-1 or TGF beta-2 in combination with bFGF exerted synergistic effects on cell proliferation in articular chondrocytes obtained from the rabbit. Their results suggest a critical role of cytokine combinations in the development of articular cartilage degeneration after immobilisation (Liu et al. 2014). Thorp et al. (1992) reported on the effects of transforming growth factor-beta 1, -beta 2 and -beta 3 in cartilage and bone cells during endochondral ossification in the chick. They discovered that TGF-beta localization controlled an increase in type II collagen and mRNA expression in transitional chondrocytes, suggesting a role for TGF-beta in the induction of synthesis of extracellular matrix (Khaghani et al. 2012).

Yamashita et al. (2002) used fibroblast growth factor-2 (FGF-2) to determine severity of joint disease in Adjuvant-Induced arthritis in rats. Their results suggested that FGF-2 modulated disease progression, but did not affect initiation of the arthritis (Sefat et al. 2016).

As shown above there are many reports regarding using of various cytokines, growth factors and developed chondrocyte culture methods to stimulate the chondrocyte proliferation in vivo and in vitro. However, until now there are very few reports looking at the effect of the transforming growth factor beta family of cytokines (TGF-β1, TGF-β2 and TGF-β3) on chondrocyte cell–cell and cell–ECM adhesion, cell proliferation, migration and wound healing.

Davidson et al. (2005) investigated the change of transforming growth factor-beta signalling in cartilage of old mice and found that the TGF-β2 and 3 were reduced in 2 year old mice in comparison to 5 months old animals. The conclusion of their reports shows that the TGF-beta appears to play an important role in repair of cartilage and a lack of TGF-beta responsiveness in old mice might be at the root of osteoarthritis (Zheng et al. 2016).

Oka et al. (2007) used TGF-β2 to examine the role of TGF-β signalling in regulation of chondrogenesis and osteogenesis during mandibular development. Their data suggested that there are differential signal flows in response to TGF-β control of chondrogenesis and osteogenesis during mandibular development (Lawrence 2001).

Pohlers et al. (2007) published a paper about up-regulation of the transforming growth factor-β pathway in rheumatoid arthritis (RA). They reported that the pathogenetic role of TGF-β-induced effects on Synovial Fibroblast (SFBs) in RA (Docagne et al. 2004).

The importance of transforming growth factors in wound healing has caused significantly increase in research on these types of cytokines.

Related work was also carried out by Mark Ferguson and O’Kane (2004) who identified some TGF-β1 related therapeutic targets. They found that the growth factor profiles differ significantly in embryonic and adult tissue. For example, levels of TGF-β1 and TGF-β2 in embryonic wounds are very low, however the levels of TGF-β3 is much higher in comparison to adult wounds (Van der Kraan and Van den Berg 2008). Similarly, Gorvy et al. (2005) found that by inhibiting TGF-β1 and TGF-β2, whilst simultaneously increasing TGF-β3 concentrations in adult wounds, near embryonic repair could be achieved in adults (Yao et al. 2003).

Noticeably significant research on chondrocyte adhesion is lacking. In this work cell response in terms of cell phenotype, adhesion, alignment, wound healing capacity and secretion of extracellular matrix molecules will be examined in relation to exposure to TGF-β2 in multiple 2 dimensional (Multilayer 3D) culture systems.

## Materials and methods

### Primary chondrocyte culture

Six neonate Sprague–Dawley rats were euthanized according schedule-1 of euthanasia. The Hyaline cartilages from knee joints were harvested, and their extracellular matrix was digested for 45 min at 37 °C using 15 ml Trypsin EDTA-0.25% and 10 ml Collagenase type-II (Sigma Aldrich, UK). The cell suspension was centrifuged at 550 g to separate the cells from solution. The supernatant was discarded and the cell pellte were re-suspended in 5 ml-high glucose Dulbecco’s modified eagle’s medium (DMEM) (Sigma Aldrich, UK) with 1% l-glutamine supplementation. In 5 ml cell suspension 14 × 10^5^ cells were counted using a haemocytometer, giving a cell density of 2.8 × 10^5^ cells/ml. The cell suspension was kept in a 15 ml centrifuge tube and incubated at 37 °C until preparation of a TGF-β2 solution.

Addition of this growth factor to the culture media facilitated the investigation of the effects of TGF-β2 on the chondrocyte cell length, proliferation rate, cell adhesion and on wound repair of the created wound model.

All results from each part of the experiment were standardised using the setting of the initial cell density as 100% and then comparing the remaining cells with initial percentage. Standard error (± SE) is also used to estimate any error in measurements and to determine the accuracy of the results.

### Reconstitution of TGF-β2

TGF-β2 is a multifunctional peptide soluble in acid solvent. It is also soluble in water; however, the solution would be sticky and difficult to aliquot and utilise. It is also evident that TGF-β2 requires a carrier molecule such as BSA to enable cell uptake by cell (Spagnoli et al. 2007). Several TGF-β2 with different concentrations (5, 10 and 50 ng/ml) were used previously in our research while measuring the percentage of wound closure for chondrocyte mono and multilayers. Therefore a stock solution of 5 µg/ml TGF-β2 was made by dissolving 2 µg of TGF-β2 in 400 µl of sterile 4 mM HCl containing 1 µg/ml BSA (Spagnoli et al. 2007; Zhang et al. 2007).

### Primary chondrocyte culture

The experiment consisted of five tissue culture flasks labeled as Control, HCl, HCl/BSA, BSA and TGF-β2. A total of 2.88 × 10^5^ cells per culture flask were subjected to this set of experiments. By resuspending this amount of cells into 5 ml cell culture media, each culture flask is supposed to have a cell density of 5.76 × 10^4^ cells/ml.

**Fig. 1:**
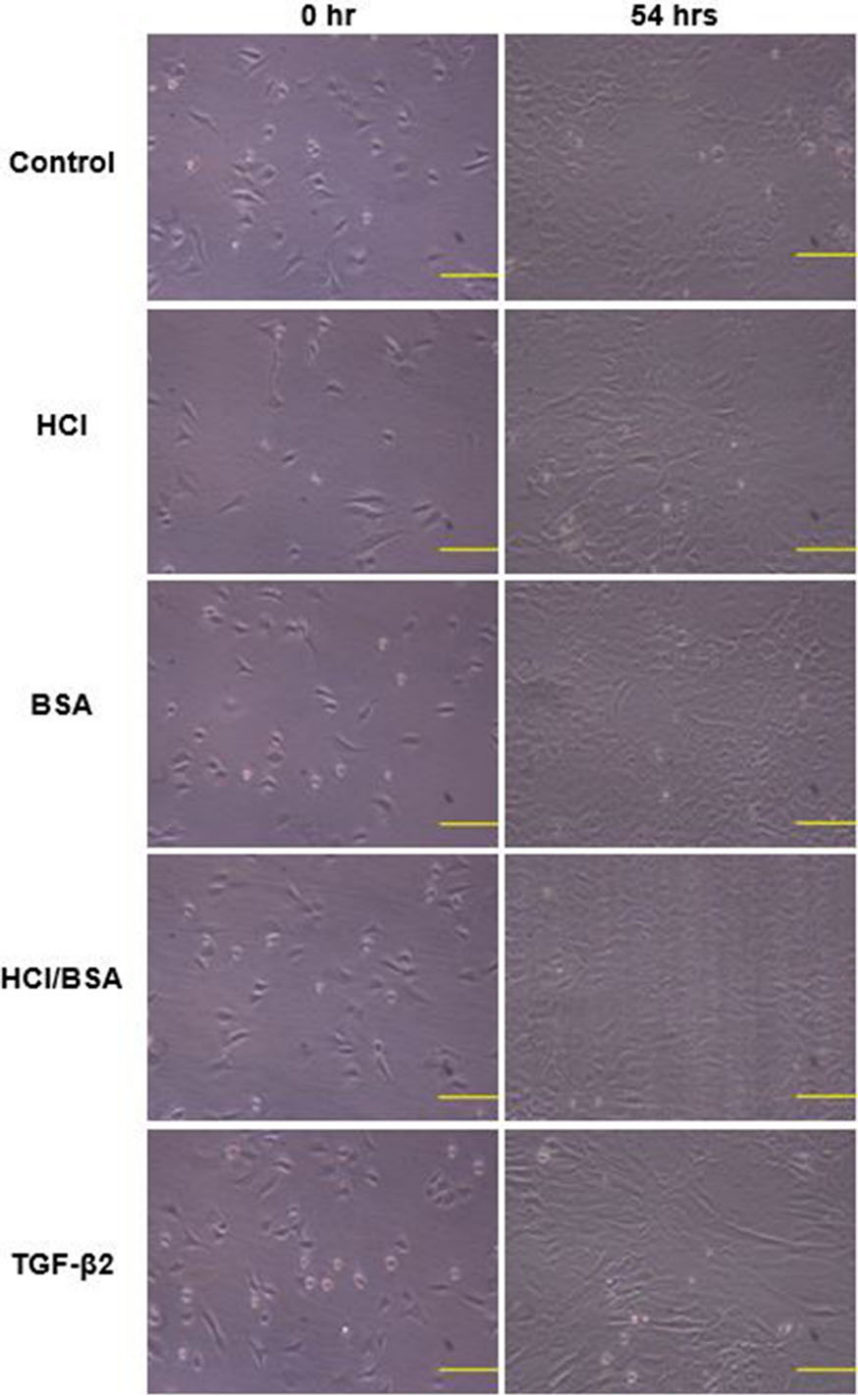
The chondrocytes cultured in media with different supplementations. The captured images are the first and last pictures (scale bar 50 μm)

A total of 5 ml of cell suspension were poured into the culture flask labeled as control. A total of 10 µl of 4 mM HCl was added into another 5 ml cell suspension labeled as HCl. 10 µl of BSA solution, dissolved in sterilised distilled water with a concentration of 1.5 µg/ml, added into 5 ml cell suspension and poured into the cell culture flask labeled as BSA. Another 5 ml cell suspension was supplemented with 10 µl of 4 mM BSA/HCl prepared earlier and transferred into the culture flask named as BSA/HCl. The final 5 ml cell suspension was supplemented with 10 µl TGF-β2 solution, poured into the culture flask labeled as TGF-β2 and incubated at 37 °C with another four culture flasks. Every 24 h the cell cultures were checked and imaged using a phase-contrast microscope with 10 × magnification. The images were saved for future reference. Also, every 48 h, the media were aspirated, discarded and replaced with fresh media with the same (10 µl/ml v/v) TGF-β2 supplementation (Fig. 1).

The experiment was repeated three times to obtain significant data for statistical analysis.

### Measurement of cell length

Cell-length measurements of all cell cultures supplemented with BSA, BSA/HCl, control and TGF-β2 were performed 12 h after cell seeding into the tissue culture flasks. This time allowed for primary chondrocytes settling and attaching to the solid surface.

Recording of the cell length continued every 24 h. A total of 30 randomly selected cells were imaged and saved and their lengths measured using NIH Image J software. As the primary chondrocytes were isolated from different zones of cartilage they had different shapes, and hence the largest dimension of the cells was considered as the cell length. The average data coming from repeated experiments were calculated and set as average chondrocyte cell length. The mean cell length and standard deviation with error bars were calculated using Microsoft Excel. The standardised cell lengths from each treatment were compared with the standardised dimension of chondrocytes in the control culture to find out whether or not TGF-β2 causes any change in the length of the cells.

Following standardisation of the data, the first measurements were set as 100% and the subsequent data were determined as percentage average.

### Wound closure assay

All five chondrocyte cultures reached 100% confluency after 132 h.

The wound repair capacity of multilayer primary chondrocyte culture was investigated using model wound closure assay. A wound model was created by scratching the cell layer using a fine-tip extended transfer pipette of 1 mm diameter (Sigma Aldrich, UK). Following scratching, wound width was measured via Image J software every 2 h at 10 different points along the width until the wound was totally healed except for the model wound treated with TGF-β2, which did not fully heal during the experiment. The re-identification of these points was achieved by drawing 10 lines perpendicular to the scratch in the cell layer on the underside of the culture flask with an alcohol-resistant marker pen. Measurements of wound width were then taken to the right of the point at which each perpendicular line intersected with the model wound. An average wound width of ~ 131.77 µm was recorded and the normalized % model wound closure was used to eliminate different wound width along the wound bed, for future analysis regarding the effect of different growth factor.

### Primary chondrocyte detachment analysis

The strength of primary-chondrocyte attachment on substrate in the presence of different supplementations was analysed using the trypsinisation assay.

Trypsin is a temperature-dependent digestive enzyme which breaks down the cell–extracellular matrix and cell–cell binding proteins at the carboxyl end (Sigma Aldrich, UK). The cleavage of adhesion proteins results in cell detachment from substratum and/or separation from other cells.

The assay was carried out at room temperature of 18 °C. The media were aspirated from the tissue culture flask and the flask was rinsed three times with Hank’s balanced salt solution (HBSS) to remove any serum from the confluent layer of cells. After aspiration of the last HBSS, 2 ml of trypsin was poured into the culture flask to detach the cells from the culture flask and their ECM. The protein digestion process was imaged by phase-contrast microscope, with a digital camera installed on the microscope, and Image J (NIH) software. A sequence of 60 images with 10-s time intervals were recorded and saved for further analysis.

A total of 2 ml of serum containing media was added to the cell suspension immediately after detachment of the cell from the culture flask. After trypsinization, the suspension was used for cell count.

The time taken for the chondrocytes to get a round shape and shiny appearance, signs of detachment, was recorded.

**Fig. 2:**
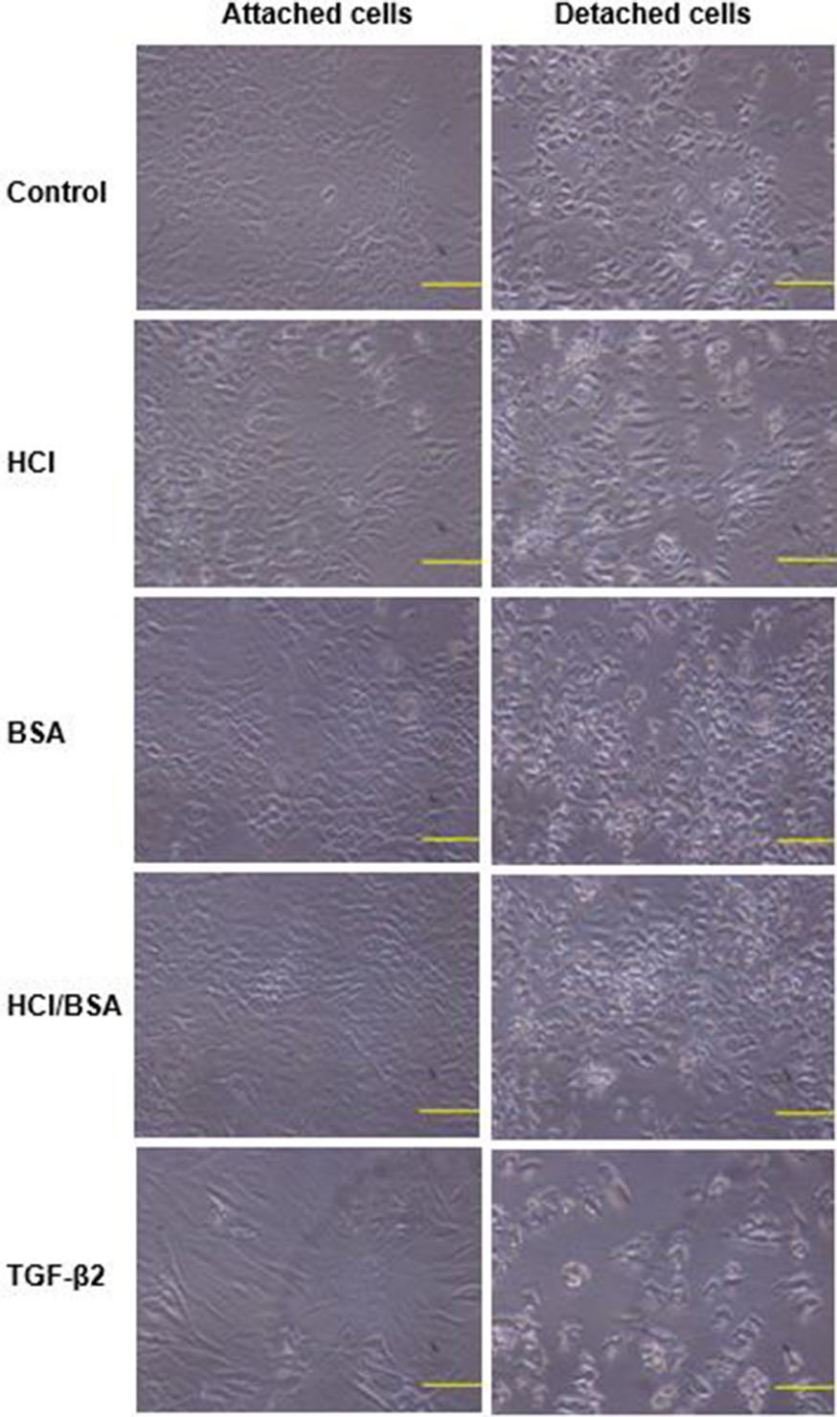
Trypsinisation assay before (left) and after (right) detachment (scale bar = 50 μm)

The recorded time was multiplied by 10, which was the time interval, and then divided by 60 to obtain the detachment time per second. The time taken for the detaching of a cell in the control culture was converted to percentage average as 100% and the other cell cultures, containing different supplementations, were compared against the control. Microsoft Excel was used to calculate the mean, standard deviation and a graph was then plotted to demonstrate % detachment time for each supplementation (Fig. 2).

The final suspensions obtained from trypsinisation were used to evaluate cell proliferation rate. This proliferation rate was achieved by counting the trypsinized cells and comparing with initial seeded cells.

### RNA extraction

Total RNA was extracted from primary chondrocyte cultures using TRIzol Reagent (Invitrogen, UK). Samples (2 µg) were treated with DNaseI and revers transcribed in a 25 µl volume reaction using Superscript II enzyme (Invitrogen, UK) and random hexamer primers. Products were diluted 1:100 and stored at − 20 °C until used for RT-PCR (real time -PCR).

### RT-PCR

Primers were used at 5 µM with 10 µl of SYBR Green Master Mix (Thermo Fischer, UK) in final volume of 20 µl under the following condition: 95 °C for 15 min (denaturation), 45 cycles at 94 °C for 15 s, 60 °C for 20 s (annealing) and 72 °C for 20 s (amplification). PCR amplification and real-time fluorescence detection were performed by the Light Cycler version 1.0 detection system (Roche). Relative expression was calculated according to the 2^−Δ Δ*C*t^ method (Livak and Schmittgen 2001) (Table 1).

**Table 1:** Utilized primers for RT-PCR amplification

Gene	Forward primer	Revers primer
Smad2	5′-GCTGTTTTCCTAGCGTGGCTT-3′	5′-TCCAGACCCACCAGCTGACT-3′
Smad3	5′-GCATCAGCCGCTTCTCAAGT-3′	5′-ATCTCCCCACCATCACCTCC-3′
Smad4	5′-CCTTCTGGAGGAGATCGCT-3′	5′-TCAATGGCTTCTGTCCTGTGG-3′
Smad7	5′-AATGTGTTTTCTAGATTCCCAACTTCTT-3′	5′-CACTCTCGTCTTCTCCTCCCAGTA-3′
TGF-β2	5′-CAGCGCTACATCGATAGCAA-3′	5′-GGTCCTCAGTGTAGCCCAAG-3′
TβRI	5′-TTAAAAGGCGCAACCAAGAAC-3′	5′-GTGGTGATGAGCCCTTCGAT-3′
TβRII	5′-GACATCAATCTGAAGCATGAGAACA-3′	5′- GGCGGTGATCAGCCAGTATT-3′

### Western blot analysis

Cells were rinsed, and scrapped in RIPA lysis buffer (25 mM TrisHCl pH 7.6, 150 mM NaCl, 1% NP-40, 1% sodium deoxycholate, 0.1% SDS, Thermo Scientific, UK), supplemented with phosphatase and protease inhibitors (Thermo Scientific Halt Protease Inhibitor Cocktail, Halt™ Phosphatase Inhibitor Cocktail). The extracts (50 μg protein) were subjected to fractionation in 10% SDS-PAGE, transferred to polyvinylidene fluoride membranes (Kinesis, UK), and reacted with TβRI, TβRII (1:500), Smad2/3 or phospho-Smad2/3 polyclonal antibodies (1:1000) (Thermo Fisher Scientific, UK). Blocking buffer was added to each membrane and 0.5 µg/ml Pierce Goat anti-Rabbit IgG Secondary Antibody Horseradish Peroxidase (HRP) (Thermo Fisher, UK) was diluted in the blocking buffer (1:3000). The signals were revealed with SuperSignal West Pico Chemiluminescent Substrate (Thermo Fisher Scientific, UK) and exposed to X-ray film. The membranes were also reacted with anti β-actin to verify equal loading.

### Statistical analysis

The effect of different supplementations in chondrocyte cell proliferation, cell length, adhesion, and wound repair was statistically analysed by One-way ANOVA test. This test was performed to evaluate the statistically significant difference between the means of the treatments. The difference between the means of the treatments was assumed as Ha, and the similarity was assumed as H_0_.

$${\text{H}}_{0} :\;{\text{Mean}}1 = {\text{Mean}}2 = {\text{Mean}}3 = {\text{Mean}}4$$

Statistical significance was determinated by student’s test. *p* values less than 0.05 were considered significant.

## Results

Primary chondrocytes, cultured in multilayer in high-glucose DMEM media supplemented with 10% FCS, were used as control culture.

**Fig. 3:**
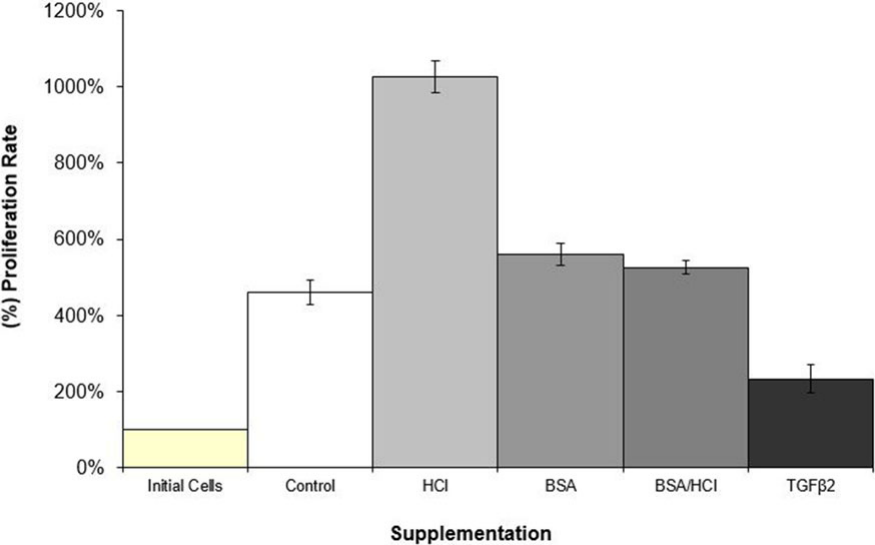
Graph of primary chondrocyte proliferation cultured in DMEM media in various supplementations. Initial cell concentration, which was 2.8 × 10^5^ cells/ml, was set as 100%

The evaluation of the effect of different supplementations in chondrocyte proliferation showed that the HCl induced cell proliferation at the highest level (unpublished work) with 1026.23% increase (Fig. 3).

**Fig. 4:**
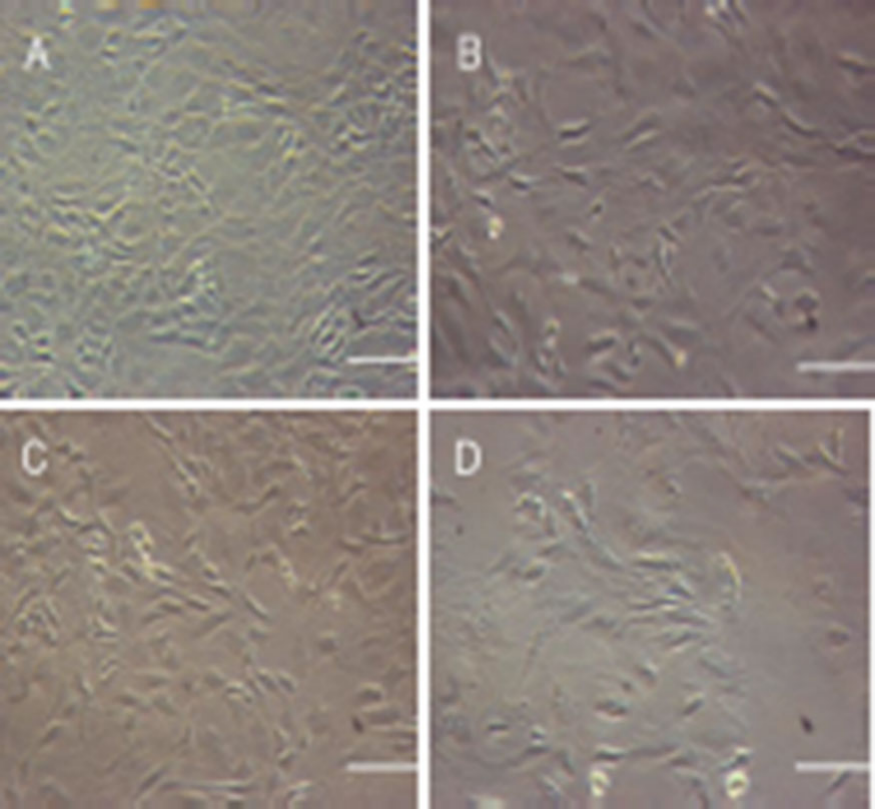
Culture of chondrocyte cells in high glucose DMEM with different supplementations: **a** HCl; **b** BSA; **c** BSA/HCl, and **d** BSA/HCl/TGF-β2 (scale bar = 50 μm)

In contrast, TGF-β2 induced apoptosis rather than proliferation. The cell density was lowered at 50.7% when compared with control and the proliferation rate was 2.33-fold. Unlike TGF-β2, HCl induced cell proliferation at the highest level, which was 2.23-fold more than control (Figs. 3, 4).

The cells treated with TGF-β2 developed a well-spread fibroblastic shape acquiring a mean length of 14.20 μm in diameter. This indicates that TGF-β2 increases excessive synthesis of ECM proteins conclusion which are involved in the formation of fibroblast-type morphology and consequently dedifferentiation of chondrocyte in vitro. In comparison, HCl (12.2 μm ± 0.002 SE), BSA (11.18 μm ± 0.002 SE), BSA/HCl (10.45 μm ± 0.002 SE) had no effect on cell length and cells under these treatment regimens resembled in the control treatment group (12.51 μm ± 0.002 SE). There was no recognisable difference in cell morphology between HCl, BSA, BSA/HCl and control (Fig. 4).

**Fig. 5:**
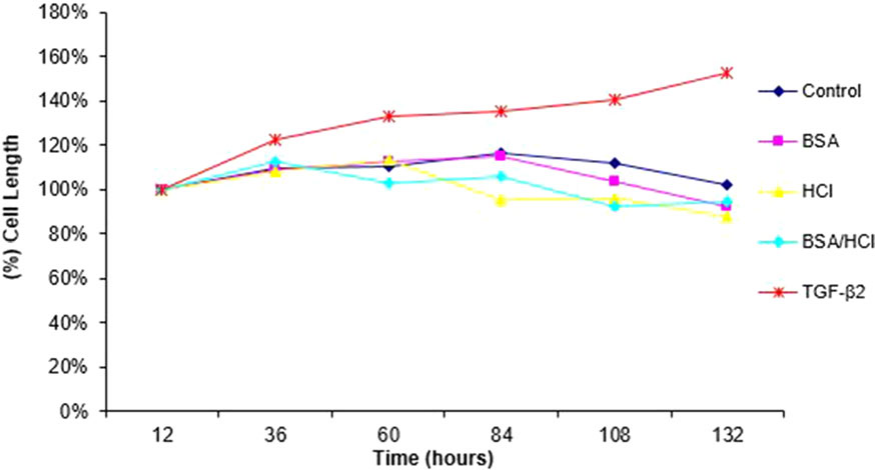
Graphs of primary chondrocyte cell length during 132-h culture with various supplementations with standard error bar

Another four culture environments with addition of HCl, BSA, BSA/HCl and TGF-β2 were compared against the control culture (Fig. 5).

Interestingly, each cell culture reached its largest cell length after different times.

TGF-β2 increased the chondrocyte cell length up to 152.9% over a period of 132 h. This could be related to an increase in the production of components of the ECM, which in turn, via up or down regulation of specific integrins, induced changes in chondrocyte shape. It is well known that the cytoskeleton determines the cell shape by anchoring to the integrin via the actin filament. ELISA could determine the type of integrin binding to the ECM/ligands. In contrast, the smallest cell length was observed in BSA/HCl contained media with 112.85% increase and 10.45 μm. The average change of cells in their widest dimension in control was 115.97% with 12.51 μm. Only chondrocytes cultured in the TGF-β2 contained medium demonstrated an almost constant increase in cell length with all others showing irregular changes. This is because of low-proliferation capacity of chondrocyte in the presence of TGF-β2 as revealed in determination of the effect of TGF-β2 on cell proliferation. The initial cell density was set as 100% and all other cultures were compared with this initial setting. The proliferation rate of control culture was 460.35%, corresponding to a 4.6-fold increase in cell density at the end of the experiment.

**Fig. 6:**
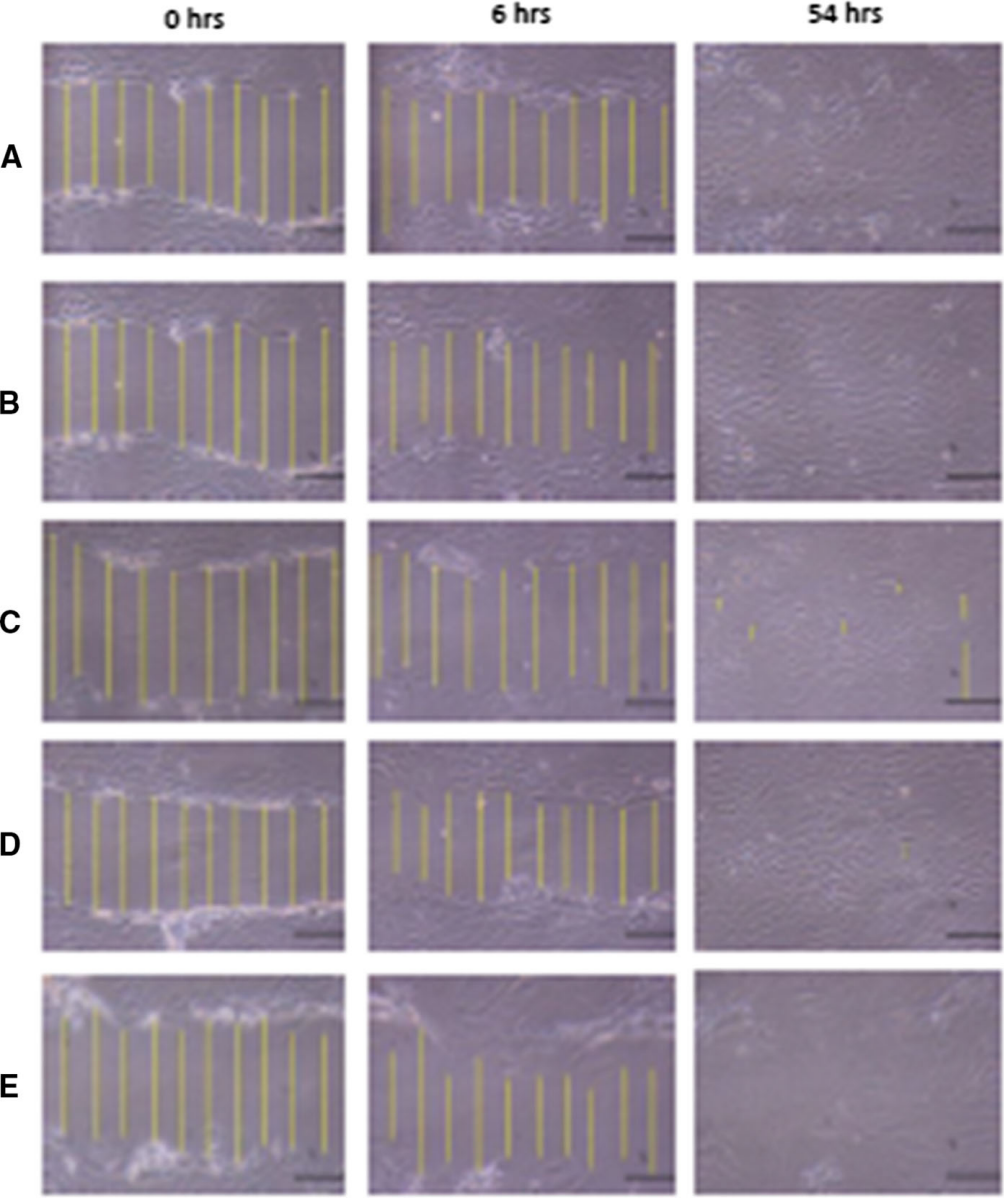
Microphotographs of wound closure assay for primary chondrocyte: **a** control; **b** HCl; **c** BSA; **d** BSA/HCl, and **e** TGFβ-2. Multilayer cultures were scratched by tip of a plastic pipette of 1 mm and measured using image analysis software. An average wound size of ~ 131.77 µm was recorded after initial scratch at 0 h (scale bar = 50 μm)

During 54 h model wound closure assay, only in controls and HCl contained culture was the gap completely closed (Fig. 6).

The fastest wound healing occurred in an acidic medium with 10 μl 4 mM HCl. The application of BSA/HCl/TGFβ-2 and the application of BSA containing media appeared to inhibit wound closure. Initially, cell migration was detected circa 6 h after scratching. This may suggest that the chondrocyte cell requires a revitalisation time after mechanical stress in the form of scratching.

**Fig. 7:**
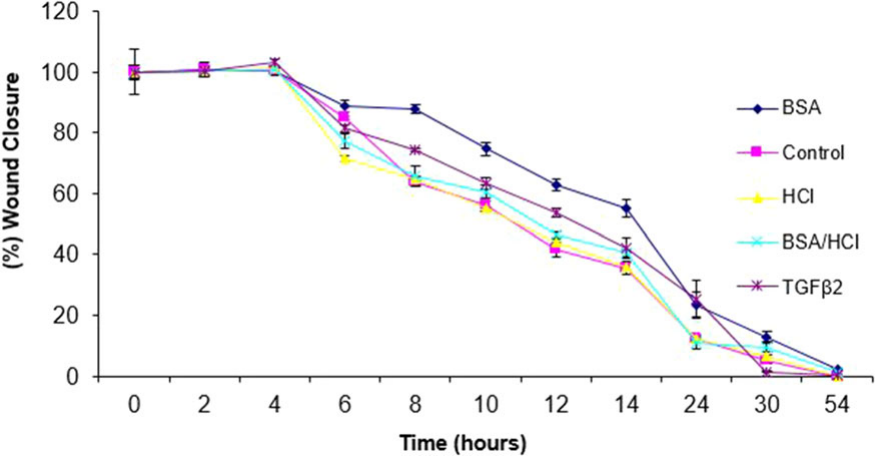
, Graph of wound closure for primary chondrocytes cultured in BSA, BSA/HCl, HCl, and TGF-β2 contained media and control

The results of model wound closure assays were set as a normalized percentage average. The outcomes were used to plot a graph to demonstrate the comparison of the wound closures of chondrocytes cultured in multilayer with various supplementations. It can be seen that the wound models of control and HCl contained cultures were closed at a slower rate after 54 h and those supplemented with BSA (2.88 µm ± 0.4 SE), BSA/HCl (1.38 µm ± 0.4 SE), and TGF-β2 (0.33 µm ± 0.1 SE) closed completely, after 54 h but at a faster rate, with almost complete closure (wound closure ~ 98%) after ~ 30 h (Fig. 7).

Results from trypsinisation assay demonstrated the time required for detachment of chondrocyte cells from substrate and separation from each other. This time shows the strength of cell-substrate and/or cell–cell adhesion (Fig. 2).

Chondrocytes cultured in the presence of 1% v/v of 4 mM HCl were detached in 7.778 min, which corresponds to being 6.87% longer than control culture. This time for TGFβ-2 was the lowest with 5.056 min, which caused the cells to detach 2.22 min faster than the cells in the control culture. The cells in BSA and BSA/HCl contained mediums showed recognisable reductions in detachment time of 5.94 and 6.05 min, respectively.

It can be seen that TGF-β2 addition resulted in lower % detachment (~ 70%) whereas the HCl showed a similar result to control followed by BSA and BSA/HCl additions with ~ 85% detachment.

The above set of experiments revealed that TGFβ-2 has an inhibitory effect on chondrocyte cell proliferation and reduces cell density in the presence of 10% FCS in planar culture system. This type of growth factor induces an increase in cell length by synthesis of extracellular matrix proteins and by stimulating the cell to acquire fibroblastic morphology. Excessive ECM molecules capture a large area in the culture flask and consequently cause the cells to spread well in multilayer with weak cell adhesion ability. In contrast, 1% v/v 4 mM HCl contained media promoted cell proliferation up to 10.12-fold, which is very close to the required cell density for ACI.

The results of statistical analysis revealed that the null hypothesis was rejected in all tests, as in all tests the F > Fcrit and the *p* < 0.05, except for wounds repair, which showed that the model wounds on the chondrocyte multilayers with different supplementations were almost similar in terms of wound closure response even after 54 h (Fig. 7).

**Fig. 8:**
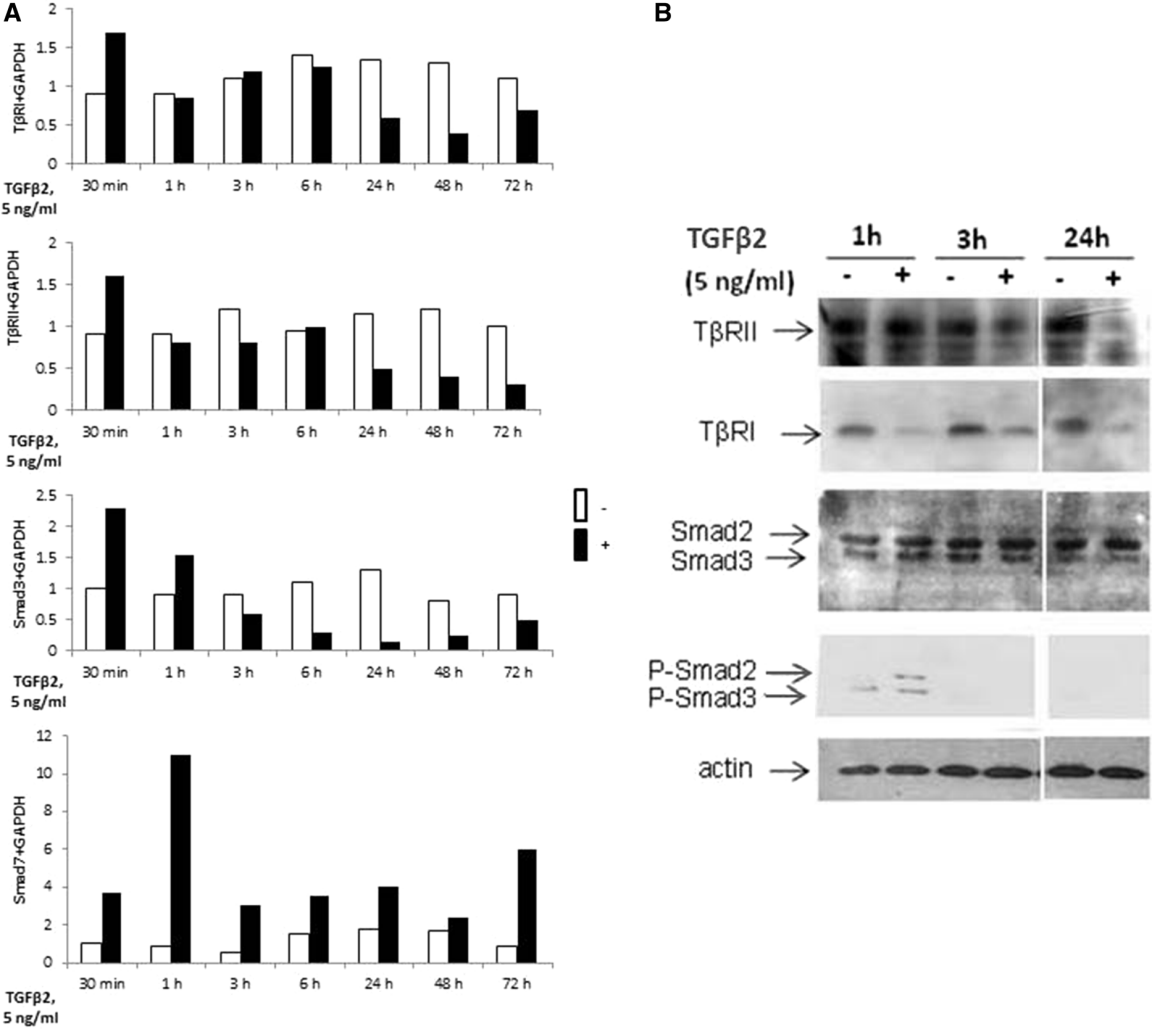
Effect of TGF-β2 on expression of TβRI, TβRII, Smad2/3, actin and phosphorylated Smad2/3 protein analysed by RT-PCR and western blot technique. **a** Articular cartilage chondrocytes were incubated with 5 ng/ml TGF-β2 for different times. At the end of incubations, TβRI, TβRII, Smad3 and Smad7 mRNA levels were assayed by RT-PCR. **b** In addition, TβRI, TβRII, Smad2/3 and phosphorylated Smad2/3 protein expression were analyzed by western blot analysis. As control for equal loading, β-actin was also run in each blot. The modulation of mRNA expression was expressed as the percentage of controls after normalisation to the GAPDH signal

We investigated the effect of TGF-β2 on mRNA expression of TGF-β signalling genes in a dose-dependent manner, using real-time RT-PCR. A 48-h incubation with TGF-β2 significantly reduced the expression of both TGF-β receptors and Smad3, whereas the Smad7 mRNA level was increased. No significant effect was observed on Smad2 and Smad4 (Fig. 8a).

A time-course study revealed that, at mRNA levels, TGF-β2 quickly upregulates its own receptors and Smad3, since it increases their expression as soon as 30 min of treatment. For longer treatments, TGF-β2 exerted the opposite effect and downregulated TGF-β receptors (after 24 h of incubation) as well as Smad3 (after 3 h of incubation). Independent from incubation time the expression of Smad7 was upregulated by TGF-β2 (Fig. 8a).

Furthermore, western blot analysis showed that TβRII is downregulated after 24 h whereas TβRI protein expression is decreased as soon as 1 h after TGFβ2 treatment. In addition, as expected, TGF-β2 induced Smad2/3 phosphorylation. Expression of actin filament was slightly increased at the presence of TGF-β2 (Fig. 8b).

Modifications of gene expression under TGF-β2 treatment could be due to an increased degradation rate and/or a reduced transcription. Inhibition of de novo transcription clearly showed that TGF-β2 reduced the mRNA half-life of both TGF-β receptors. Indeed, the TβRI half-life is about 20 min but was reduced to 10 min when chondrocytes were incubated with TGF-β2, and the TβRII mRNA half-life is 45 min for control cells and was reduced by almost 80% after TGF-β2 treatment.

## Discussion

Cartilage, a highly specialized tissue, sustains compressive load by cushioning the bones at joints, and by providing almost frictionless condition for joints to reduce wear. Cartilage as an avascular tissue has poor repair capacity and thus any damage or degradation on this tissue can cause pain, complexity in mobility and adversely affect daily life.

Monolayer culture of the primary chondrocyte resulted in dedifferentiation of cells and production of stress fibers. This characteristic was prevented by 3D chondrocyte culture (Multilayer Culture).

To our knowledge, so far, there are only few surgical options for treatment of chondral or osteochondral lesions of the hip. One such treatment is that of ACI which has been used frequently for treatment of knee cartilage defects with good outcomes, but there is probably only one or no use of ACI for the hip. It is our recommendation that collagen (type-I) gel (or patches) be used for prior chondrocyte implantation.

Another application of this research output/findings is 3D scaffold design which is a critical and essential part of tissue engineering. Our results from detachment assay suggested clearly that TGF-β2 will decrease mechanical strength and biological interactions of chondrocyte culture, but it could have different effect when manipulated with other proteins.

TGF-β2 increased cell length of chondrocyte, but decreased its proliferation rate and the wound healing process. TGF-β2 also decreased cell adhesion ability to the surface of the culture flask which could be a sign of decrease in cell anchoring agent/ligands and proteins required to cell–cell and cell–ECM adhesion.

Our research shows that TGF-β2 exerts a differential effect on the transcription of genes implicated in the Smads pathway. While TGF-β2 upregulates its receptors and Smad3 for short incubation (at least at mRNA level), it downregulates them in the long term. In addition, it upregulates Smad7 and does not significantly alter Smad2 and Smad4 expression. This positive and negative feedback loop of the TGF-β2 pathway induces differential response of chondrocytes to TGF-β2. The mechanisms responsible for modulation of Smads and for TGF-β receptor expression seem to be different.

We have established that, in primary chondrocytes, TGF-β2 acts, at least in part, by strongly decreasing the mRNA stability of its receptors. This rapid turnover potentially allows the receptor rate to change rapidly in response to its own ligand. We cannot, however, exclude the possibility that TGF-β downregulates its receptors also at the transcriptional and translational levels.

Concerning Smad effectors, we established that the downregulation of Smad3 mRNA expression in TGF-β2 treated chondrocytes was not due to decreased transcript stability, suggesting a transcriptional effect of TGF-β2.

In contrast to Smad3, Smad7 mRNA expression was rapidly and markedly induced by TGF-β2. Increased expression of the inhibitor Smad7 has been associated with inhibition of TGF-β signaling. Smad7 could negatively regulate TGF-β signaling; on one hand by inhibiting R-Smad activation by TβRI or by enhancing TβRI degradation in the cytoplasm, and on the other hand by disrupting the formation of the TGF-β induced functional Smad-DNA complex in the nucleus (Zhang et al. 2007). These TGF-β2 induced modifications on expression of TGF-β receptors and Smads may participate in the chondrocyte-phenotype changes observed in a pathology associated, at least in the first stage, with an increase in the TGF-β2 level.

## Conclusions

This work clearly demonstrated a successful and typical cell engineering of chondrocyte multilayers with our own model using isolated primary RAC by cell seeding, multiple 2D cell expansion and differentiation in vitro and without the use of bioreactor. Therefore, the same model can be used for isolation of HAC from clinically approved human biopsies for autologous chondrocyte implantation, and/or multilayer production for, e.g. cytotoxicity tests. Our model can also be used for direct expansion of the limited number of chondrocytes obtained from RAC or HAC, reaching higher densities similar to those used in conventional cartilage tissue engineering based on cells expanded by 2D cultures.

The effect of manipulated TGF-β2 with other growth factors and/or proteins needs to be investigated to finalize the utilization of this growth factor and design of scaffolding in treatment of different types of arthritis.

## Abbreviations

IL-1: Interleukin-1
TGF-β: Transforming growth factor-β
GDF-5: Growth and differentiation factor-5
ePPi: Extracellular inorganic pyrophosphate
PKC: Protein Kinase C
R-Smads: Receptor-regulated Smads
BSA: Bovine serum albumin
bFGF: Basic fibroblast growth factor (bFGF)
FGF-2: Fibroblast growth factor-2
ECM: Extra-cellular matrix
RA: Rheumatoid arthritis
SFBs: Synovial Fibroblasts
DMEM: Dulbecco’s modified eagle’s medium
HBSS: Hank’s balanced salt solution
RT-PCR: Real time-polymerase chain reaction
FCS: Fetal calf serum
ELISA: Enzyme-linked immunosorbent assay
ACI: Autologous chondrocyte implantation
RAC: Rat articular chondrocytes (RAC)
HAC: Human articular chondrocyte (HAC)
